# Quantitative Proteomic Profiling of Fungal Growth, Development, and Ochratoxin A Production in *Aspergillus ochraceus* on High- and Low-NaCl Cultures

**DOI:** 10.3390/toxins13010051

**Published:** 2021-01-13

**Authors:** Yan Wang, Yue Guan, Wei Lin, Hao Yan, Jing Neng, Peilong Sun

**Affiliations:** 1 College of Food Science and Technology, Zhejiang University of Technology, Hangzhou 310014, China; wangyan062006@zjut.edu.cn (Y.W.); zjutgy2020@163.com (Y.G.); linwei5913@163.com (W.L.); nengjing@zjut.edu.cn (J.N.); 2 Key Laboratory of Food Macromolecular Resources Processing Technology Research (Zhejiang University of Technology), China National Light Industry, Hangzhou 310014, China; 3 Zhejiang Provincial Center for Disease Control and Prevention, Hangzhou 310051, China

**Keywords:** proteomics, ochratoxin A, *Aspergillus ochraceus*, salt, food safety

## Abstract

Dry-cured meat products are worldwide food with high-salt content, and filamentous fungi are beneficial to the maturation process. However, some salt-tolerant strains of *Aspergillus* and *Penicillium* produce ochratoxin A (OTA) on these products and thus threaten food safety. In our study, proteomic analysis was performed to reveal the mechanism of adaptability to high-salt environment by *Aspergillus ochraceus*. Twenty g/L and 70 g/L NaCl substrates were used to provide medium- and high-NaCl content environments, respectively. The NaCl addition could induce fungal growth, but only 20 g/L NaCl addition could induce spore production while 70 g/L repressed it. Proteomics analysis identified 2646 proteins in *A. ochraceus* fc-1, of which 237 and 251 were differentially expressed with 20 g/L and 70 g/L NaCl addition, respectively. Potential factors affecting fungal growth and development were identified by GO and KEGG analyses of biological process, cellular component, and molecular function terms. The results revealed that ergosterol synthesis pathway was significantly upregulated with 20 g/L and 70 g/L NaCl addition. However, fungal growth and development including OTA production were complex processes associated with many factors including nutrient uptake, cell membrane integrity, cell cycle, energy metabolism, intracellular redox homeostasis, protein synthesis and processing, autophagy, and secondary metabolism. Reactive oxygen species may be an important window to understand the mechanism that medium-salt content was conducive to intracellular signal transduction while high-salt content caused oxidative stress. The findings would help to improve the processes and storage conditions of dry-cured meat products.

## 1. Introduction

In many food-processing processes, especially in the processing of meat products, the addition of table salt is necessary, which can improve the flavor and texture and can reduce water activity and inhibit the growth of microorganisms. Dry-cured meat products are worldwide high-salt-content food, and molds contribute to the development of the sensory qualities. However, some filamentous fungi of *Aspergillus* and *Penicillium* are able to grow on these NaCl-rich substrates and produce Ochratoxin A (OTA) [1].

OTA is a potent nephrotoxic mycotoxin [2,3] and is classified as a Group 2B compound (a possible human carcinogen) by the International Agency for Research on Cancer. OTA, which was produced by *P. verrucosum*, *P. nordicum*, *A. westerdijkiae,* and *A. ochraceus*, was detected in dry-cured meat products including dry-cured sausages and hams [4,5]. However, the contamination of OTA in these food commodities received less attention and few countries and organizations established a maximum permissible level of OTA content. One μg/kg of OTA was limited in fresh pork and derived meat products in Italy [1].

Medium- or high-salt concentration often accelerates the mycelial growth of *P. verrucosum* and *P. nordicum*, but the influence of salt on the OTA production remains, many argue, depending on the different types of strains. NaCl addition is usually in favor of OTA production [6]. In addition, OTA production may be beneficial for *Penicillium*’s adaption to NaCl-rich substrates [7]. In recent studies, natural preservatives (spices) and biocontrol (*Debaryomyces hansenii*) have shown potential of controlling OTA production by *Penicillium* in high-NaCl-content meat model [8,9].

However, similar studies about *Aspergillus* remain limited. *A. ochraceus* could grow at low water activities and some strains even grow strongly at 37 °C, while its closely related species, *A. westerdijkiae,* would not grow at this temperature [10,11]. With increasing global warming, threat from *A. ochraceus* would become more considerable. *A. ochraceus* was likely to have greater growth rates in a medium supplied with certain concentrations of NaCl (0–80 g/L), and the colony diameter was the largest at the salt content of 40 g/L. The capability of OTA production was inhibited when salt content was 40 g/L in *A. ochraceus* [12]. The knowledge of the mechanism about adaption to high-NaCl environments by *Aspergillus* would help for OTA control in dry-cured products.

Proteomics is a powerful tool for determining the mechanisms of biochemistry for how *A. ochraceus* conform in NaCl-rich environments [13]. In this study, isobaric tags for relative and absolute quantification (iTRAQ)-based quantitative proteomic analysis were performed to research proteome changes in high- and low-NaCl conditions. The potential factors affecting fungal growth and development were identified based on GO (Gene Ontology) and KEGG (Kyoto Encyclopedia of Genes and Genomes) analyses of biological process, cellular component, and molecular function terms.

## 2. Results

### 2.1. Influence of NaCl Addition on Mycelial Growth and Sporulation Ability of A. ochraceus

**Figure 1:**
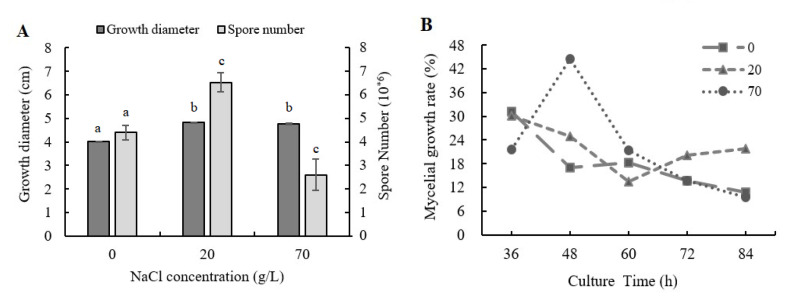
The mycelial diameter and spore production amounts after four days of culture with NaCl addition. (**A**) Mycelial diameter and spore number were measured after four days of culture in PDA medium with NaCl addition. (**B**) Growth rates were measured in four days of culture in PDA medium with NaCl addition. Results are presented as the mean of three repetitions. Different letters indicate a significant difference between the corresponding values (*p* < 0.05).

With the addition of NaCl, the mycelial growth and the sporulation ability of *A. ochraceus* were significantly changed. The influence of NaCl addition on the fungal mycelial growth is shown in Figure 1A. The mycelial diameter was 20.1% and 18.6% increased when 20 and 70 g/L NaCl were added compared to control, respectively. As shown in Figure 1B, the growth rates at 36, 72, and 84 h were lower than control samples when 70 g/L NaCl was added, while the growth rates were much higher at 48 h and 60 h. The mycelial growth was significantly accelerated in culture times of 36–60 h and it resulted in the final length of the mycelial being larger than the control group when 70 g/L NaCl was added. The growth rate was higher than 20% except the value at 60 h when 20 g/L NaCl was added. However, the mean growth rate of control samples was 18.1%, and it indicated that 20 g/L NaCl addition could stably promote fungal growth.

The influence of NaCl addition on the fungal sporulation ability is shown in Figure 1A. The capacities of spore producing were 48.4% increased with 20 g/L NaCl addition and were 41.0% decreased with 70 g/L NaCl addition, respectively.

### 2.2. Influence of NaCl Addition on OTA Production of A. ochraceus

**Figure 2:**
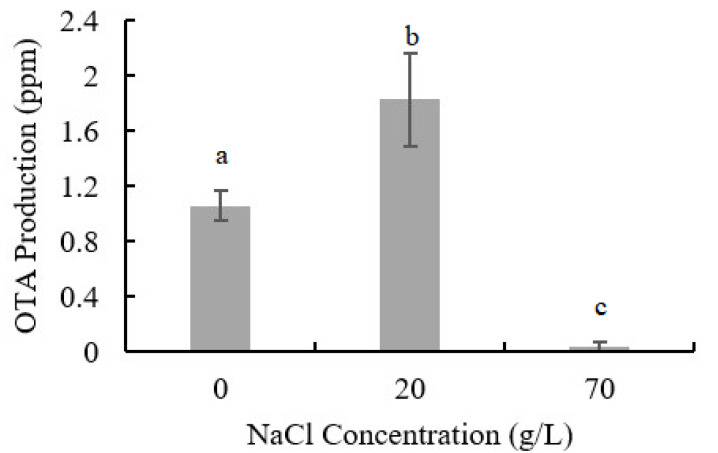
OTA production in seven days of culture in PDA medium with NaCl addition. Results are presented as the mean of three repetitions. Different letters indicate a significant difference between the corresponding values (*p* < 0.05).

The influence of NaCl addition on OTA production of *A. ochraceus* is shown in Figure 2. Low concentration of NaCl could promote OTA production, while higher concentration inhibits it; 1.827 ppm OTA was detected with 20 g/L NaCl addition (72.7% higher than control samples) and only 0.033 ppm OTA was detected in the samples with 70 g/L NaCl addition.

OTA content is an important indicator to measure the safety of foods such as cereals and dry-cured meat products, and our results indicated that low concentration of NaCl may increase the risk of OTA accumulation. Proteomics analysis was further performed to reveal the mechanism of fungal growth, development, and OTA production in environments of low- or high-NaCl concentrations.

### 2.3. Alteration of Proteins with or without NaCl Addition

**Figure 3:**
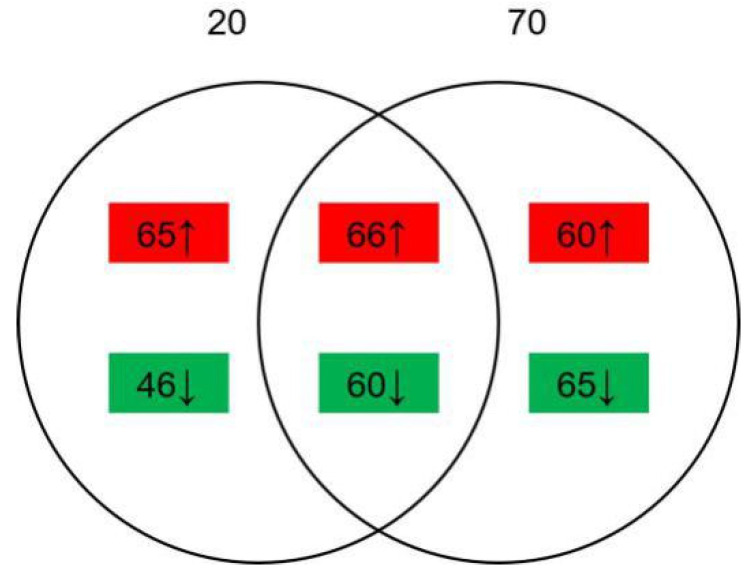
Identified differentially expressed proteins (DEPs). Red or ↑ represents significantly upregulated proteins and green or ↓ represents significantly downregulated proteins. Group A, 20 g/L NaCl addition vs. control; Group B, 70 g/L NaCl addition vs. control.

The comparison between the samples with 20 g/L NaCl added or without was named group A, while that with 70 g/L NaCl added or without was named group B. A total of 2646 proteins were identified. There were 237 differentially expressed proteins (DEPs) identified (|Log_2_ ratio | > 1.5, *p* < 0.05), of which 131 were upregulated and 106 were downregulated in group A; 251 DEPs were identified (|Log_2_ ratio| > 1.5, *p* < 0.05), of which 126 were upregulated and 125 were downregulated in group B. As shown in Figure 3, 126 proteins were found both regulated in two groups, of which 66 proteins were upregulated and 60 proteins were downregulated. The alternation number of proteins in group B was larger than group A, indicating that 70 g/L NaCl addition may cause more influence on the growth and development of *A. ochraceus* than does 20 g/L NaCl addition.

### 2.4. Hierarchical Clustering, Functional Classification, and Enrichment Analysis of DEPs

Hierarchical cluster analysis was performed on DEPs, and the results are shown in Supplementary Figure S1. The clustering results of target proteins can help us distinguish protein subsets with different expression patterns from protein collections. Proteins with similar expression patterns may have similar functions or participate in the same biological pathway.

**Figure 4:**
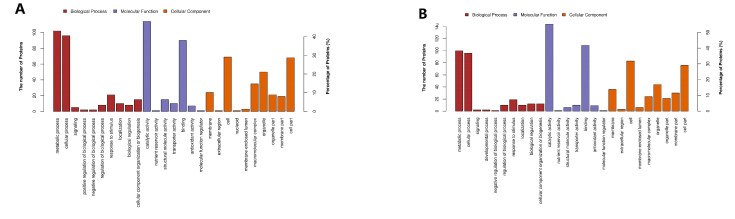
GO functional classification of DEPs. (**A**) Group A, 20 g/L NaCl addition vs. control; (**B**) Group B, 70 g/L NaCl addition vs. control.

GO functional classification was shown in Figure 4. In group A and group B, the functions of most DEPs were related to catalytic activity, binding, structural molecule activity, transporter activity, and antioxidant activity. Most DEPs participated in metabolic processes, cellular processes, response to stimuli, localization, biological regulation, cellular component of organization or biogenesis, and other important biological processes. Notably, the amounts of DEPs related to catalytic activity in group B were much larger than group A.

**Figure 5:**
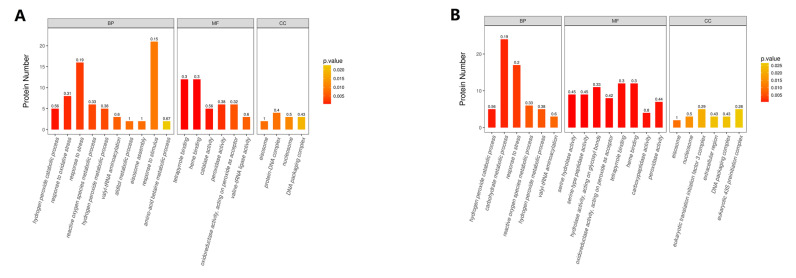
GO enrichment analysis of DEPs. The enrichment value represents the ratio of the number of DEPs vs. the total number of identified proteins annotated to the GO functional category. (**A**) Group A, 20 g/L NaCl addition vs. control; (**B**) Group B, 70 g/L NaCl addition vs. control.

Enriched GO terms are shown in Figure 5. GO enrichment analysis identified tetrapyrrole binding, heme binding, catalase activity, hydrogen peroxide catabolic process, response to oxidative stress, peroxidase activity, response to stress, reactive oxygen species’ metabolic process, hydrogen peroxide metabolic process, and valyl-tRNA aminoacylation activity that underwent significant changes in group A, while serine hydrolase activity, serine-type peptidase activity, heme binding, tetrapyrrole binding, carboxypeptidase activity, peroxidase activity, antioxidant activity, hydrogen peroxide catabolic process, catalase activity, and carbohydrate metabolic process showed significant changes in group B.

**Figure 6:**
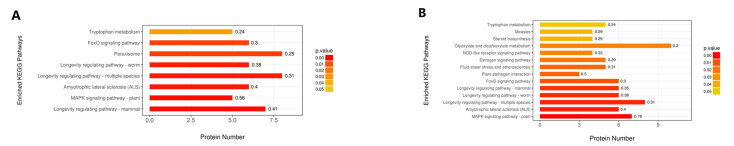
KEGG enrichment analysis of DEPs. The enrichment value represents the ratio of the number of DEPs vs. the total number of identified proteins annotated to the KEGG pathway category; (**A**) 20 g/L NaCl addition vs. control; (**B**) 70 g/L NaCl addition vs. control.

KEGG pathway enrichment results are shown in Figure 6. KEGG pathway enrichment analysis indicated that proteins involved in MAPK (mitogen activated protein kinase) signaling pathway, peroxisome, longevity regulating pathway, FoxO signaling pathway, and tryptophan metabolism were significantly regulated in two groups. However, proteins related to estrogen signaling pathway, NOD (nucleotide-binding and oligomerisation domain-containing protein)-like receptor signaling pathway, glyoxylate and dicarboxylate metabolism, and steroid biosynthesis were significantly regulated in group B.

### 2.5. Classification of DEPs

Because of the limited studies on *A. ochraceus* and the database with relatively few relevant entries, some functions of DEPs remain uncharacterized. Even so, many DEPs were selected and classified into categories of extracellular hydrolase, ergosterol synthesis pathway-related enzymes, cell cycle-related proteins, energy metabolism-related enzymes, non-phosphorylated oxidoreductases, antioxidant defense system-related proteins, protein synthesis-related proteins, chaperones, autophagy -elated proteins, and secondary metabolite synthase, as shown in Table 1.

These proteins participated in the biological process of nutrient uptake, maintaining integrity of the cell membrane, cell cycle, energy metabolism, maintaining intracellular redox homeostasis, protein synthesis and processing, autophagy, and secondary metabolism.

## 3. Discussion

Depending on the different processing technique, dry-cured meat products have a high-salt content, even above 5% NaCl. Due to a low water activity in these products with high-NaCl content, they are not susceptible to pathogens, but they have been found with the residue of OTA produced by *P. nordicum* and *A. ochraceus*. In addition, cheese also has a nearly 1.7% NaCl content and could be influenced by OTA contamination. The factor was taken into consideration, and then 20 or 70 g/L NaCl (2%, 7%) was chosen as the study concentration for proteomics analysis. Notably, *A. ochraceus* could grow at low water activities and high temperature. Thus, *A. ochraceus* could be a potential threat especially in the maturation process of dry-cured meat products in summer conditions [14]. The mechanism of adaption for high-salt environment by *A. ochraceus* was studied through proteomics analysis for further improvement of process and storage conditions of these products.

### 3.1. Fungal Growth Promoted by NaCl Addition

Our results indicated that NaCl addition was beneficial for the growth of *A. ochraceus*, and lower concentration of NaCl addition induced sporulation while high concentration repressed it.

Fungi can produce a series of extracellular enzymes, especially hydrolases, which helps them obtain nutrients from the surrounding environment [14]. Our results confirmed the influence of NaCl on nutrient uptake of *A. ochraceus*. The neutral protease 2, secreted lipase, and endoglucanase 3 were 2.10, 2.03, and 1.74 times upregulated in group B, while the neutral protease 2 was 1.72 times increased in group A. Upregulation of extracellular enzymes may accelerate the fungal growth. However, 70 g/L NaCl addition saw more acceleration on the secretion of extracellular enzymes than did 20 g/L.

**Figure 7:**
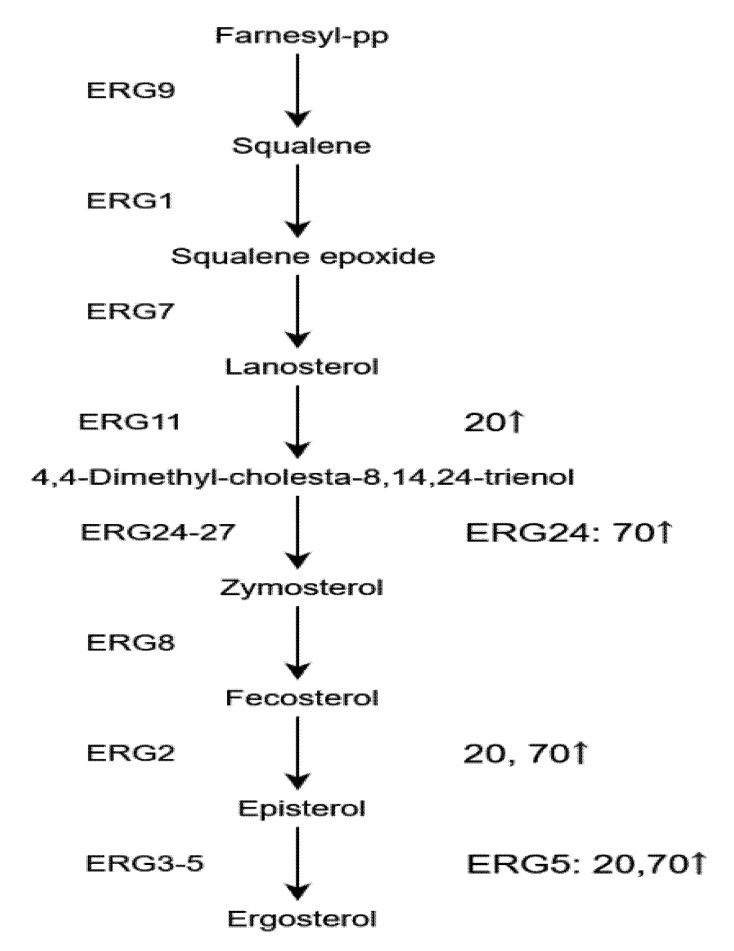
Specific ergosterol biosynthetic pathway and related enzymes are shown. 20: Group A, 20 g/L NaCl addition vs. control. 70: Group B, 70 g/L NaCl addition vs. control. ↑: upregulation.

As a component of cell membranes, sterols may affect various functions of cell membranes, such as maintaining the permeability and fluidity of cell membranes [15]. Ergosterols are found almost solely in fungi, enriched in the plasma membrane, and could be used as an indicator of fungal biomass. Four enzymes involved in ergosterol biosynthesis pathway (Figure 7) were detected as DEPs (Ergosterol ERG2, 5, 11, and 24). ERG11, ERG2, and ERG5 were significantly upregulated in group A, while ERG24, ERG2, and ERG5 were significantly upregulated in group B. NaCl could upregulate the ergosterol biosynthesis pathway both in the samples with 20 and 70 g/L NaCl addition and the increased content of ergosterol could be an intuitive evidence for the acceleration on the fungal growth [16]. Further on, it may be an important mechanism for maintaining permeability of cell membrane under NaCl stress.

Cell cycle-associated proteins (cell cycle arrest protein (BUB3), DNA helicase (MCM6) and serine/threonine-protein phosphatase 2A regulatory subunit A (PP2A)) were 2.02, 1.61, and 1.53 times upregulated when exposed to 20 g/L NaCl, respectively. The results were consistent with the promoting effect of 20 g/L NaCl addition on fungal growth and spore production.

### 3.2. Energy Metabolism and Oxidative Stress Influenced by NaCl Addition

Energy metabolism was significantly affected when NaCl was added and the content of the enzymes related to glycolysis, tricarboxylic acid cycle, and respiratory chain were changed. Glucose-6-phosphate isomerase and glyceraldehyde-3-phosphate dehydrogenase were decreased in both groups. Fructose-bisphosphate aldolase and triosephosphate isomerase were 1.57 and 1.52 times upregulated in group A, while phosphoglycerate mutase showed downregulation. Notably, in group A, downregulation of glyceraldehyde-3-phosphate dehydrogenase and upregulation of triosephosphate isomerase may induce the higher production of dihydroxyacetone phosphate and, thus, induce the higher production of glycerol, which was benefit for fungal growth under NaCl stress [17]. However, triosephosphate isomerase showed no significant changes when more NaCl was added, which may be due to the overall downregulation of energy metabolism in group B. Nevertheless, it was difficult to reveal the influence from 20 g/L NaCl addition on overall energy metabolism through analysis of glycolysis related enzymes.

Proteins related to tricarboxylic acid cycle were significantly downregulated, including two malate dehydrogenases (0.39; 0.60) and aconitate hydratase (0.61) in group B. Malate dehydrogenase could be a metabolic longevity regulator in yeast [18]. Downregulation of malate dehydrogenase could be an intuitive evidence of cell aging in the samples with 70 g/L NaCl addition. However, no significant changes of these proteins were found in group A.

Reactive oxygen species (ROS) are not only important to signal transductions but also cause cellular damages, depending on the concentration. The correct redox balance plays a crucial role in fungal growth, conidial formation, and secondary metabolism [3]. The respiratory chain could be an important source of ROS within mitochondria. The respiratory chain mainly includes four complexes, of which complexes I (NADH dehydrogenase) and Ш (cytochrome bc1 complex) are the main sources of reactive oxygen species [19]. Notably, no significant changes of complexes I- and Ш-related proteins were found in group A. However, two kinds of putative NADH-ubiquinone oxidoreductase (0.56; 0.6) subunits were significantly downregulated and the cytochrome bc1 complex subunit was 1.71 times upregulated in group B. These results indicated that 70 g/L NaCl addition may accelerate the production of ROS within mitochondria through respiratory chain.

Moreover, other parts of respiratory chain, even including ATP synthase, were also affected by NaCl addition. Succinate dehydrogenase assembly factor 2 (0.65) was decreased when 70 g/L NaCl was added. Four (1.85, 1.68, 1.58, 1.57) and three (3.67, 1.75, 1.72) kinds of cytochrome c oxidase subunits were significantly upregulated in group A and B, respectively, while cytochrome c oxidase subunit 1 (0.51) showed downregulation in group B. Two and four kinds of ATP synthase subunits were decreased when 20 and 70 g/L NaCl were added, respectively, and one kind of ATP synthase subunit alpha was 1.71 times upregulated in the samples with 20 g/L addition. Downregulation of ATP synthase in the samples with 70 g/L NaCl addition represented the downregulation of energy metabolism directly. However, 20 g/L NaCl addition more slightly influenced the energy metabolism than did 70 g/L NaCl addition.

ROS are generated in several cellular systems and mitochondria may not be the main source of cellular ROS [19]. ROS can be produced by xanthine dehydrogenase in cytosol, cytochromes P450 and protein disulfide isomerase in endoplasmic reticulum, and phagocyte NADPH oxidase in plasma membrane and other oxidoreductases. The addition of NaCl increases the production of these kinds of proteins (using FAD^+^, NAD^+^, or NADP^+^ as an electron acceptor), including cytochrome b5, sulfite reductase, thioredoxin, and protein disulfide isomerase. Cytochrome b5 is a type of cytochrome P450 enzyme system that can participate in biological processes such as fatty acid synthesis, sterol synthesis, and xenobiotic metabolism. The putative cytochrome b5 (A0A2I2GRW8) was 1.53 times increased when 20 g/L NaCl was added, while it was 4.10 times increased when 70 g/L NaCl was added, indicating that NaCl addition may cause disruption of fungal redox balance and higher concentration of NaCl addition made a bigger impact than did a lower concentration.

Protein disulfide isomerase and endoplasmic reticulum-resident protein were involved in the formation of disulfide bonds in proteins in the endoplasmic reticulum. Oxidative equivalents flowed into substrate protein from endoplasmic reticulum-resident protein through direct dithiol-disulfide bond exchange between protein disulfide isomerase and endoplasmic reticulum-resident protein [20]. Two protein disulfide isomerases (1.77 and 1.52 times in group A, while 2.72 and 1.66 times in group B) were significantly upregulated in two groups and 70 g/L NaCl addition seemed to have a larger influence on the proteins related to formation of intramolecular disulfide bonds than did 20 g/L NaCl addition.

Antioxidative defense system proteins were also influenced by NaCl addition and that could be an evidence that NaCl addition can cause fungal oxidative stress. Peroxidase, superoxide dismutase, catalase, and glutathione S-transferase were significantly upregulated in both groups. In detail, peroxidase (A0A2P2H6U1) was 1.54 and 1.72 times upregulated in group A and B, respectively. Peroxidase (A0A1L9S160) was 1.67 times upregulated with 70 g/L NaCl addition. Superoxide dismutase was 2.18 and 3.80 times upregulated in group A and B, respectively. Five kinds of catalase (3.32, 2.18, 1.87, 1.79, and 1.71 times in group A, while 5.24, 3.69, 2.69, 1.93, and 2.63 times in group B) were significantly changed by NaCl addition. Notably, the fold changes in group B were much larger than fold changes in group A. The results indicated that there was much more accumulation of ROS in the sample with 70 g/L NaCl addition than in 20 g/L NaCl addition and they conformed to the regulation of ROS production-related proteins listed above.

In brief, 20 g/L NaCl addition produced moderate ROS and induced the fungal growth and development, while higher concentration caused over accumulation of ROS and was harmful, especially in fungal development.

### 3.3. Protein Synthesis and Processing Repressed by NaCl Addition

Protein synthesis is directly related to ribosomes. Our results showed that ribosome biosynthesis was repressed by the presence of NaCl. RNA polymerase I subunit beta (0.62) was significantly downregulated in the samples with 70 g/L NaCl, while no significant changes were found in the sample with 20 g/L NaCl. Moreover, nucleolar proteins (cbf5, nucleolar protein 58, nucleolar GTP-binding protein 2 and dbp3) related to pre-rRNA processing and most of the ribosomal proteins were downregulated in two groups. The 40S ribosomal protein S14 and ribosomal protein L24 were significantly upregulated in group A, while other ribosomal proteins were repressed in two groups. Actually, the synthesis of rRNA and ribosomal proteins were coupled and downregulation of RNA polymerase I would repress pre-rRNA processing and ribosome assembly [21].

Aminoacyl-tRNA is crucial for ribosome assembly and translation. Aminoacyl-tRNA synthetase was repressed when exposed to NaCl, including seryl-tRNA synthetase (0.65 times in group A, while 0.64 times in group B), valyl-tRNA synthetase (0.60, 0.66 times, and no significant changes in group A, while no significant changes, 0.63, and 0.66 times in group A), and lysyl-tRNA synthetase (0.58 times in group B). The effect of downregulation on the aminoacyl-tRNA synthetase was proportional to the NaCl concentration and the cofactor for methionyl-and glutamyl-tRNA synthetase (0.56) was downregulated in group B.

Ribosome biosynthesis is a key process of growth and constitutes a major consumer of cellular resources. This pathway is very tightly regulated to correct ribosome biosynthesis with a wide variety of environmental and metabolic change, and intracellular insults. Downregulation of ribosome biosynthesis could be an adaption for insufficient nutritional metabolism under NaCl stress, especially in higher concentration because of the downregulation of RNA polymerase I in group B. Notably, oxidative stress represses protein synthesis by inhibiting different sub-steps of the ribosomal elongation cycle [22]. Much more accumulation of ROS in the sample with 70 g/L NaCl could be a reason for inhibiting protein synthesis.

Interestingly, pre-mRNA splicing in two groups was overall upregulated. small nuclear ribonucleoprotein (SnRNP) assembly factor was 1.73 and 1.97 times increased in the sample with 20 and 70 g/L NaCl addition, respectively. Two pre-mRNA splicing factors were upregulated and another was downregulated by the presence of 20 g/L NaCl. Translation initiation factor and elongation factor were also decreased, except translation initiation factor 1A (1.61 and 1.96 time in group A and B, respectively) in two groups. The downregulation of translation initiation factor would directly decrease protein synthesis, especially in group B. Interestingly, translation initiation factor 1A is associated to abiotic stress response in *Tamarix hispida* [14]. Similar mechanism may exist in *A. ochraceus* but further study is needed.

After protein synthesis in ribosomes, peptide chain folding process was performed to form the native conformation. ATP-dependent molecular chaperones’ heat shock proteins 90 (Hsp90) and 70 (Hsp70) are employed for protein folding and remodeling to maintain protein homeostasis [23]. Aqueous-exposed hydrophobic domains of client proteins are bound with Hsp70 and Hsp90 to assist sequestration of hydrophobic residues in the core of the proteins [24,25].

Our results showed that the abundances of chaperones were greatly changed with NaCl addition. Four kinds of Hsp70 including BIP (0.67 and 0.51 times in group A and B, respectively) were significantly downregulated in group B, while two kinds of Hsp70 were significantly downregulated in group A. The Hsp90-domain-containing protein was significantly downregulated in two groups and HtpG was decreased in the samples with 70 g/L NaCl addition. However, downregulation of some chaperones may not directly cause the change of the majority relative protein concentration, because the additional workload caused by the decrease could be buffered by the capacity of other chaperones [26].

In general, protein synthesis was significantly affected, and higher oxidative stress in group B would seriously influence peptide synthesis in ribosomes. However, the specific influence of NaCl addition on protein processing remained unclear.

### 3.4. Signal Transduction and Autophagy Activated by NaCl Addition

Fungal Ca^2+^ signaling pathway and autophagy were found affected when NaCl was added.

Ca^2+^ signaling pathway-related proteins including outer mitochondrial membrane protein porin (VDAC2) and calmodulin (CALM) were significantly regulated, especially in group B. VDAC2 and CALM were 1.87 and 1.82 times changed in the samples with 70 g/L NaCl addition, indicating the disorder of cellular Ca^2+^ signaling pathway. Actually, the influence of NaCl on cellular Ca^2+^ is predictable because of the presence of Na^+^/Ca^2+^ exchangers and competition between the extracellular Ca^2+^ and Na^+^ [27].

BIP (Hsp70) was a regulator for Ca^2+^ homeostasis in endoplasmic reticulum (ER) and could induce autophagy when unfolded/misfolded protein was in over accumulation [28]. Aspartic endopeptidase (PEP2) and saccharopepsin (PEP4) were aspartic-type endopeptidases as a component of mTORC1 (mammalian target of rapamycin) complex, associated with autophagy. PEP2 (1.66) and PEP4 (2.43) were found upregulated in the sample with 70 g/L NaCl addition. The finding confirmed the nutrient starvation condition of *A. ochraceus* when exposed to 70 g/L NaCl. However, 20 g/L NaCl addition would not induce the expression of PEP2 and PEP4.

### 3.5. Fungal Secondary Metabolism Affected by NaCl Addition

In secondary metabolism, backbone enzymes are preformed to form carbon backbones using central metabolites, that is, polyketide synthases (PKSs) and terpene synthases assembling acyl-CoAs, and NRPSs (non-ribosomal peptide synthetase) link-up amino acids. Then, tailoring enzymes, such as methyltransferases, p450 monooxygenases, hydroxylases, and epimerases, are subsequently used for further modification.

Our result showed that farnesyl pyrophosphate synthetase 1 was decreased in the samples with 70 g/L NaCl addition, and nonribosomal peptide synthetases (NRPS) 10 was 1.70 times increased in the samples with 20 g/L NaCl addition. In the previous study, *AootaA* (PKS) was 10.5 and 0.4 times changed with 20 and 70 g/L NaCl addition, respectively, while *AootaB* (NRPS) was 2.5 and 0.6 times changed [12]. The results were consistent with proteomics analysis that 20 g/L NaCl addition may induce secondary metabolisms while 70 g/L NaCl addition repressed it.

## 4. Conclusions

The NaCl addition could induce fungal growth, but only low-NaCl concentration could induce spore production while high-NaCl concentration repressed it. Comparative proteomics analysis of *A. ochraceus* with 20 and 70 g/L NaCl addition was performed in this paper, and the results revealed significant perturbation of proteins involved in nutrient uptake, cell membrane integrity, cell cycle, energy metabolism, intracellular redox homeostasis, protein synthesis and processing, autophagy, and secondary metabolism, including repression of OTA production by *A. ochraceus* depending on its concentration. More extracellular hydrolase for adaption of nutrient starvation was produced, caused by downregulation of energy metabolism, especially in higher concentration of NaCl addition. The main difference was due to the intracellular ROS. Higher ROS concentration was harmful to protein synthesis and even caused autophagy when exposed to higher concentration of NaCl. Meanwhile, secondary metabolism including OTA production was induced when 20 g/L NaCl was added and repressed when 70 g/L was added. The mechanism of adaption for high-salt environment by *A. ochraceus* was studied through proteomics analysis for further improvement of process and storage conditions of these salty foodstuffs.

## 5. Materials and Methods

### 5.1. Strains, Media, and Culture Conditions

*A. ochraceus* fc-1 was sequenced [9] and used in this work.

Potato dextrose agar (PDA; potato 200 g/L, glucose 20 g/L, agar 20 g/L) and yeast extract sucrose (YES; 20 g/L yeast extract, 150 g/L sucrose) were used. Spores were collected from 7-day-old fungal colonies grown on PDA at 28 °C. Spore suspensions’ concentrations were counted using a hemocytometer (Yuanye Bio, Shanghai, China) and changed to 10^7^ conidia/mL by 25% glycerol solution. Spore suspensions were stored at −80 °C for further research.

YES was used in proteomics research, while PDA was used to record colony diameters and detect OTA content. Culture media were prepared by adding 0, 20, and 70 g/L NaCl into PDA or YES, followed by autoclaving at 121 °C for 20 min. The cultures proceeded at 28 °C in a constant temperature incubator.

### 5.2. Mycelial Growth and Conidia Production Assessments

To evaluate the influence of NaCl on fungal mycelial growth, growth assessments were performed by adding 5 μL spore suspensions on the center of the PDA media in a petri dish. The mycelial diameters were recorded every 12 h until 84 h, and the graph was plotted according to the changes of mycelial diameter against time.

The mycelial growth rate was calculated by the following formula: growth rate (%) = (T_2_ − T_1_/T_1_) × 100%, where T_1_ is the mean colony diameter at a certain time and T_2_ is the respective value after 12 h.

The amount of conidia was counted using a hemocytometer (Yuanye Bio) after culture for four days. Briefly, five agar plugs (diameter: 8 mm) were collected from the colony, transferred into 10-mL micro reaction tubes, and 5 mL of 0.01% sterile Tween 80 aqueous solution was added. The spore suspensions were homogenized for 2 h on a rotary shaker before conidia count.

All assays were replicated in triplicate.

### 5.3. OTA Detection

For determination of OTA production, high-performance liquid chromatography (HPLC) analysis was employed. Briefly, after seven days of culture on PDA, five agar plugs (diameter: 8 mm) were collected from the colony, transferred into 2-mL micro reaction tubes, and 1 mL of methanol was added. The OTA was extracted for 2 h on a rotary shaker and the supernatants were collected, filtered through a 0.22-μm filter, and stored at −20 °C for further test.

The HPLC equipment included an Agilent 1260 series system (Agilent, Berks., UK) with a fluorescence detector and an autosampler. Analysis was performed in the isocratic mode and the mobile phase was acetonitrile:water:acetic acid (99:99:2 *v*/*v*/*v*) at a flow rate of 1 mL/min. The injection volume was 20 μL. Fluorescence detection (FLD) was used at an excitation wavelength of 330 nm and an emission wavelength of 460 nm, using a C18 column (Agilent; 150 mm × 4.6 mm, 5 μm). Pure OTA (Sigma, St. Louis, USA) was used as standard.

All assays were replicated in triplicate.

### 5.4. Proteomic Analysis

To analyze the influence of NaCl on fungal growth and development in the molecular insights, iTRAQ-based proteomic analysis was performed according to the protocol from the producer (AB SCIEX, Foster City, CA, USA). Briefly, the mycelia were collected from liquid YES media after culture for five days in a 250-mL Erlenmeyer flask, snap-frozen in liquid nitrogen, and stored at −80 °C until extraction.

SDT buffer (4% (*w/v*), sodium dodecyl sulphate, 100 mM TRIS/HCL (pH 7.6), and 0.1 M dithiothreitol) was used for protein extraction, and then bicinchoninic acid assays were used for protein quantification, followed by trypsin digestion using the filter-aided proteome preparation (FASP) method. Digested samples were desalted prior to analysis using a C18 cartridge. After lyophilization of peptides, 40 μL dissolution buffer was added and peptides were quantified spectrophotometrically at a A_280_.

Then, 100 μg of peptide was labeled for each sample. Strong cation exchange (SCX) chromatography was performed using an AKTA Purifier 100 (AKTA, Sweden) with buffer A (10 mM KH_2_PO_4_ and 25% ACN (pH 3.0)) and buffer B (10 mM KH_2_PO_4_, 500 mM KCL, and 25% ACN (pH 3.0)). For separation, the sample was loaded from the ejector onto a column equilibrated with buffer A. The gradient elution conditions were as follows: 0–25 min, 0–10% B; 25–32 min, 10–20% B; 32–42 min, 20–45% B; 42–47 min, 45–100% B; 47–60 min, 100% B; after 60 min, 0% B. A_214_ was recorded during elution, and eluted fractions were collected every minute, lyophilized, and desalted using a C_18_ cartridge.

Each fractionated sample was separated by HPLC Easy nLC (Thermo Fisher Scientific, Waltham, USA) at a nanoliter flow rate. The solvents of 0.1% formic acid (solvent A) and 0.1% formic acid acetonitrile aqueous solution (84% acetonitrile; solvent B) were used as mobile phase. The column was equilibrated with 95% solvent A. Samples were loaded from the autosampler into the loading column using an Acclaim PepMap100 (Thermo Fisher Scientific; 100 μm × 2 cm, nanoViper C_18_) and separated by an EASY analytical column (Thermo Fisher Scientific; 10 cm, 75-μm internal diameter, 3 μm, C_18_-A2) with a flow rate of 300 nL/min.

The sample was subjected to mass spectrometry using a Q-Exactive mass spectrometer (Thermo Fisher Scientific). The mass spectrometer data were acquired in positive ion mode with a selected mass range of 300–1800 m/z, and used for high-energy collisional dissociation (HCD) fragmentation. The automatic gain control (AGC) target was set to e6, the maximum injection time (IT) was 50 ms, and the dynamic exclusion time was 60 s. Polypeptides and polypeptide fragments were collected based on mass-to-charge ratio, and 20 MS2 (activation-type HCD) scans were done during each full scan. The resolution of HCD spectrum was set to 17,500 at 200 m/z, and the isolation window was 2 m/z. The normalized collision energy was 30 eV and the under fill was 0.1%.

### 5.5. Data Analysis

Statistical analyses of mycelial growth, conidia production, and OTA production were performed by Microsoft Excel (2019). Means comparison was analyzed through Duncan’s multiple-range test and *p* < 0.05 was considered statistically significant.

Proteomics analysis was performed using the Mascot search engine (http://www.matrixscience.com/; v.2.2.0) and Proteome Discoverer (Thermo Fisher Scientific; v.1.4.0). Tandem mass spectra were searched on the UniProt_*Aspergillus*_543324_20180705 database (http://www.uniprot.org/). Trypsin was designated as the cleavage enzyme, and up to two missed cuts were allowed. For precursor ions, the mass tolerance was set to 20 ppm, and for fragment ions, the mass tolerance was set to 0.1 Da. For protein quantification, the protein ratios were calculated as the median of only unique peptides for each protein, and the false detection rate (FDR) was adjusted to < 0.01. Data correction was based on the median protein ratios after normalizing of all peptide ratios, and the normalized protein median is 1.

Blast2GO (http://www.blast2go.de) was used for analysis of Gene Ontology (GO) annotation, together with BLAST searching, mapping, annotation, and InterProScan annotation. The Kyoto Encyclopedia of Genes and Genomes (KEGG) pathway database was used to predict target proteins using the KEGG Automatic Annotation Server (KAAS). Fisher’s exact tests were carried out to compare the distribution of each GO classification and KEGG pathway for the target protein set and the entire proteome, and the target protein set was subjected to enrichment analysis based on GO annotation and KEGG pathway annotation.

Protein clustering analysis was performed to standardize the quantitative information for the target protein set (−1, 1). Complex heatmap (Bioconductor; v.3.4.0) was then used to classify protein expression levels and to generate a hierarchical cluster heatmap.

All assays were replicated in duplicate. Differentially expressed proteins (DEPs) were filtered according to the significance A method [29] and *p* < 0.05.

## Figures and Tables

**Table 1:** Selected differentially expressed proteins.

Protein ID	Protein Name	Log_2_ A ^1^	Log_2_ B ^1^
Nutrient uptake			
A0A2I2GMT7	Neutral protease 2	1.72	2.10
A0A2I2GLV5	Secreted lipase	N	2.03
Q0CPJ4	Endoglucanase 3	N	1.74
Ergosterol synthesis pathway			
A0A2I2GRQ5	Cyp51A (ERG11)	1.71	N
A0A2I2G1P7	C-14 sterol reductase (ERG24)	N	2.00
A0A0U5G2H3	Putative C-8 sterol isomerase (ERG2)	1.81	1.65
A0A2I2GQM9	Cytochrome P450 (ERG5)	1.64	2.19
Cell cycle			
A0A1L9RRV9	Cell cycle arrest protein (BUB3)	2.02	N
Q5BGV2	DNA helicase (MCM6)	1.61	N
A0A1E3BQY7	Serine/threonine-protein phosphatase 2A regulatory subunit A (PP2A)	1.53	N
Glycolysis			
A0A0F8XU69	Glucose-6-phosphate isomerase	0.61	0.66
A0A2I1C3N2	Fructose-bisphosphate aldolase, class II	1.57	N
A0A254U7 × 1	Triosephosphate isomerase	1.52	N
A1CD39	Glyceraldehyde-3-phosphate dehydrogenase	0.62	N
P20445	Glyceraldehyde-3-phosphate dehydrogenase	N	0.65
A0A1R3RQC6	2,3-bisphosphoglycerate-independent phosphoglycerate mutase	0.60	N
Citric acid cycle			
Q0CIX6	Malate dehydrogenase	N	0.39
A0A1F8AGJ2	Malate dehydrogenase	N	0.60
Q2ULH0	Aconitate hydratase, mitochondrial	N	0.61
Respiratory chain			
A0A0F8WWK6	Putative NADH-ubiquinone oxidoreductase 39 kDa subunit	N	0.56
A0A2I2FTY0	Putative NADH-ubiquinone oxidoreductase subunit B	N	0.60
A0A1M3TZM0	Mitochondrial succinate dehydrogenase assembly factor 2	N	0.65
A0A2I2GQA1	Non-heme 11 kDa protein of cytochrome bc1 complex	N	1.71
A0A2G7FY34	Cytochrome c oxidase assembly protein (COX19)	1.85	3.67
A0A2I2GGG7	COX5A-domain-containing protein	1.68	N
A0A2I2GKR3	Cytochrome c oxidase subunit 7A	1.58	N
A0A1M3T952	Cytochrome c oxidase subunit 5b (COX5B)	1.57	N
A0A254TW86	COX assembly mitochondrial protein	N	1.75
A0A2I2GS70	Cytochrome c oxidase subunit	N	1.72
H6S076	Cytochrome c oxidase subunit 1	N	0.51
A0A0F8V271	ATP synthase subunit alpha	1.71	N
A0A0L1J0I5	ATP synthase subunit alpha	0.62	0.50
A0A2I2FVN7	F-type H^+^-transporting ATPase subunit g	0.65	0.64
A0A2I2G857	ATP synthase subunit beta	N	0.58
A0A1L9UVI1	Plasma membrane ATPase	N	0.46
Non-phosphorylated redox reaction			
A0A2I2G2E3	Cytochrome b5-like heme binding domain-containing protein	2.08	1.66
A0A2I2GRW8	Putative cytochrome b5	1.53	4.10
A1CEK3	Putative cytochrome b5	N	2.44
A0A1L9U588	Sulfite reductase (NADPH) hemoprotein beta-component	2.02	N
A0A2I2GR30	Assimilatory sulfite reductase	1.61	N
A0A2I2G6M7	FAD/NAD(P)-binding domain-containing protein	1.80	N
A0A0K8LSM9	Mitochondrial 5-demethoxyubiquinone hydroxylase	1.61	N
A0A2I2G2W0	Thioredoxin	1.57	2.48
A0A2I2G575	Thioredoxin-domain-containing protein	N	1.92
Q0CW86	Protein disulfide-isomerase (tigA)	1.77	2.72
G3Y6N5	Protein disulfide isomerase	1.52	1.66
Antioxidant defense system			
A0A2P2H6U1	Peroxidase	1.54	1.72
A0A1L9S160	Peroxidase	N	1.67
A0A2G7FRT8	Superoxide dismutase	2.18	3.80
A0A2I2FSX8	Cu, Zn superoxide dismutase-like protein	1.56	2.47
A0A0F8UM10	Catalase	3.32	5.24
A0A1L9RM09	Catalase	2.18	3.69
A0A0F0IKG0	Catalase	1.87	2.69
A0A2I2GCH5	Catalase	1.79	1.93
A0A2I2FWF9	Catalase	1.71	2.63
A0A1L9WU32	Glutathione S-transferase	N	1.66
Transcription, splicing and translation			
A0A2I2GRA8	DNA-directed RNA polymerase subunit beta	N	0.62
A0A2I1D678	Centromere/microtubule binding protein (cbf5)	0.60	0.54
A0A2I2GK37	Nucleolar protein 58	0.63	0.54
A0A2I2GP60	Nucleolar GTP-binding protein 2	N	0.64
A0A2I2GCA0	ATP-dependent RNA helicase (dbp3)	0.59	0.52
A0A2I2FYP3	Putative 40S ribosomal protein S14	1.58	N
A0A2I2GLK4	37S ribosomal protein S16	0.66	N
A0A1L9WPN8	Small subunit ribosomal protein S18e	0.67	N
A0A1L9PM46	Small subunit ribosomal protein S18e	0.67	N
A0A2I2FXR7	Putative 30S ribosomal subunit S4	N	0.62
A0A2I1CC93	Ribosomal protein L24	1.61	N
A0A2I2GHR7	60S ribosomal protein L37	0.41	0.58
A0A1L9RUB2	Large subunit ribosomal protein L28e	0.51	N
Q0CTP9	Large subunit ribosomal protein LP1	0.56	N
A0A1E3BC43	60S ribosomal protein L27a	0.56	0.56
A0A2I2G2I4	60S ribosomal protein L44	0.57	N
Q0CRD9	60S ribosomal protein L20	0.63	N
A0A1L9WKW1	Large subunit ribosomal protein L8e	0.63	N
A0A231MJW9	60S ribosomal protein L13	0.65	N
A0A2I2G636	Ribosomal protein L22	0.67	N
A0A2I2FUS3	Ribosomal protein L4	N	0.65
A0A1S9D4D7	Seryl-tRNA synthetase	0.65	0.64
Q5BD96	Valyl-tRNA synthetase	0.60	N
A0A2G7G5N0	Valyl-tRNA synthetase	0.66	0.63
A0A2I1C057	Valyl-tRNA synthetase	N	0.66
A0A0F0IBN1	Lysyl-tRNA synthetase, class II	N	0.58
A0A0F8UQN1	Cofactor for methionyl-and glutamyl-tRNA synthetase	N	0.56
A0A2I2GNK9	SnRNP assembly factor	1.73	1.97
A1CAI8	Putative pre-mRNA splicing factor	1.68	N
A0A1R3R8F2	Pre-mRNA-processing factor 6	1.59	N
A0A146FRE5	Pre-mRNA splicing factor	0.64	N
A0A0F0IP01	Translation initiation factor (1A/IF-1)	1.61	1.96
A0A146FRB4	Translation initiation factor (SUI1)	0.60	N
A0A017S0I0	Eukaryotic translation initiation factor 3 subunit L	0.66	0.60
A0A2I2GDK3	Eukaryotic translation initiation factor 3 subunit D	0.66	0.61
A0A2G7FVH8	Eukaryotic translation initiation factor 3 subunit A	N	0.58
A0A1L9RAX6	Eukaryotic translation initiation factor 3 subunit L	N	0.61
A0A2I2GJ17	Eukaryotic translation initiation factor 3 subunit E	N	0.64
A0A1L9UG37	Elongation factor 1-alpha	0.64	0.58
A0A2I2FT46	Elongation factor 1-beta	N	0.62
Protein folding and remodeling			
A1CEK9	Putative ER Hsp70 chaperone (BiP)	0.67	0.51
A0A017SPH2	Putative Hsp70 chaperone	N	0.63
A0A2I2FVP1	Putative Hsp70 chaperone	0.58	0.52
A0A017SGX5	Heat shock protein 70	N	0.64
A0A2I2FR82	HSP90-domain-containing protein	0.66	0.49
A0A1R3RIX4	HSP90A (HtpG)	N	0.55
Calcium signaling pathway			
A0A2J5HNT7	Outer mitochondrial membrane protein porin (VDAC2)	1.66	1.87
A0A1L9PAT2	Calmodulin (CALM)	N	1.82
Autophagy			
A0A2I2GJ12	Aspartic endopeptidase (Pep2)	N	1.66
A0A229X8Y1	Saccharopepsin (PEP4)	N	2.43
Secondary metabolism			
A0A2I2GGR1	Farnesyl pyrophosphate synthetase 1	N	0.63
Q5D0Q7	Nonribosomal peptide synthetase 10	1.7	N

^1^ Log_2_ A, Group A, 20 g/L NaCl addition vs. control; Log_2_ B, Group B, 70 g/L NaCl addition vs. control. N, means no significant changes were found or the proteins with *p* > 0.05; *p* < 0.05 for all other selected proteins.

## Supplementary Materials

The following are available online at https://www.mdpi.com/2072-6651/13/1/51/s1, Figure S1: Hierarchical cluster analysis of DEPs.
