# Supplementary figures and images for: Quantitative Proteomic Profiling of Fungal Growth, Development, and Ochratoxin A Production in Aspergillus ochraceus on High- and Low-NaCl Cultures
Source: Toxins (Basel). 2021 Jan 13;13(1):51. doi: 10.3390/toxins13010051 (PMC7828334; doi:10.3390/toxins13010051)

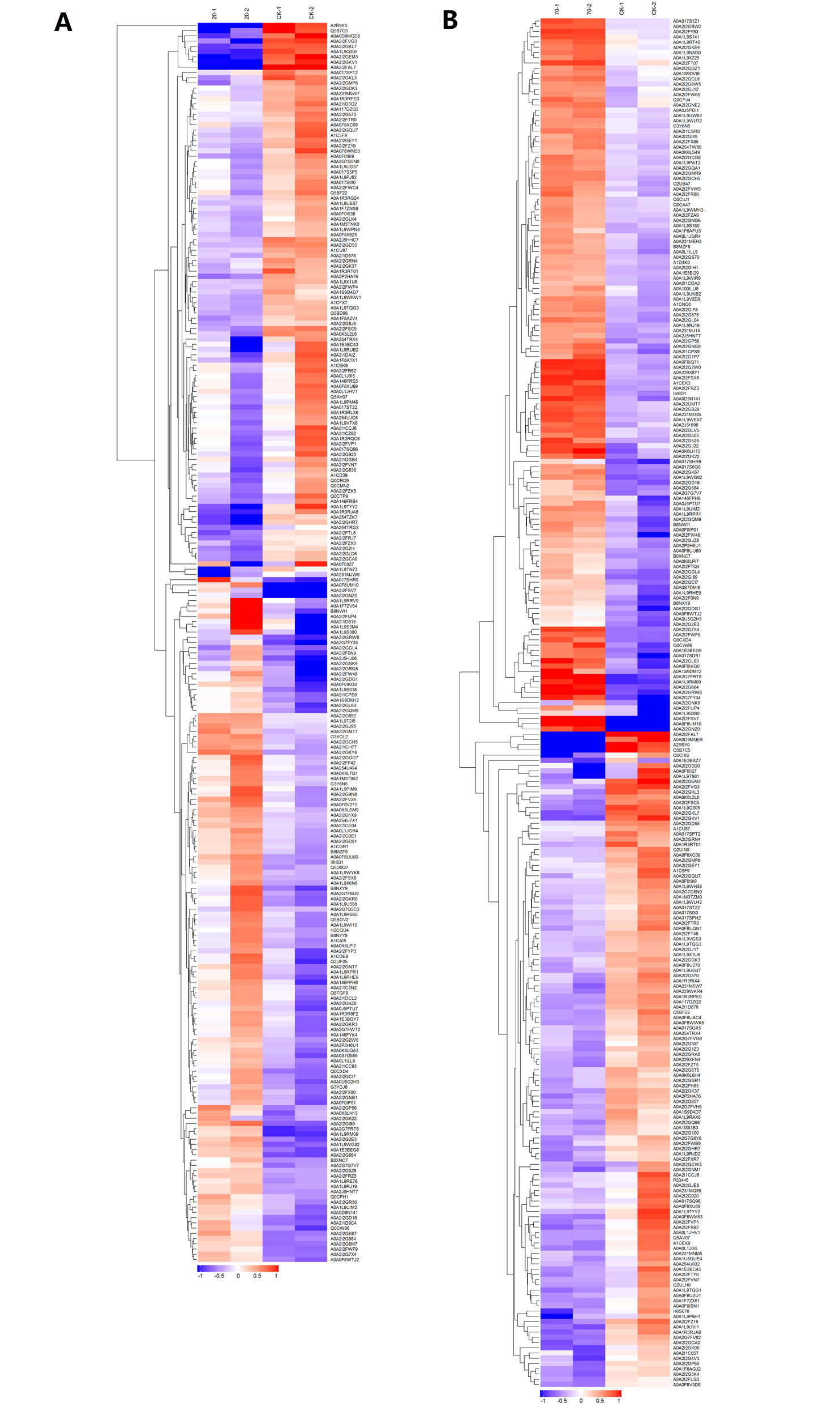

Supplement: Supplementary file 1 [file toxins-13-00051-s001.zip › Fig S1.png]
